# Eine chronische Opioidintoxikation kann zu lebensbedrohlichen Fehldiagnosen bei Palliativpatienten führen

**DOI:** 10.1007/s00482-026-00925-w

**Published:** 2026-01-27

**Authors:** Maja Falckenberg, Friedemann Nauck, Christoph Maier

**Affiliations:** 1 Das Palliativteam, Hohe Weide 17B, Hamburg, Deutschland; 2 Klinik für Palliativmedizin, Universitätsmedizin Göttingen, Georg-August-Universität, Göttingen, Deutschland; 3 Universitätsklinik für Kinder- und Jugendmedizin, Universitätsklinikum St. Josef-Hospital, Ruhr-Universität Bochum, Alexandrinenstraße 5, 44791 Bochum, Deutschland

Opioide sind unverzichtbar bei starken Schmerzen, auch im Endstadium inkurabler Erkrankungen. Sowohl zur Schmerztherapie und Behandlung von Atemnot als auch im Rahmen einer medikamentösen Sedierung gehören sie auf Palliativstationen und in Hospizen zu den am häufigsten verabreichten Pharmaka [2]. Zumindest bei vorher opioidnaiven Patienten sind hierfür niedrige Dosierungen des Opioids ausreichend [4]. Nebenwirkungen wie Obstipation, Juckreiz, Übelkeit und Erbrechen können zumeist erfolgreich behandelt werden, sodass die Opioidtherapie deswegen nicht beendet werden muss [1, 2]. Anders als die akute Ateminsuffizienz bei unsachgemäßem Vorgehen entwickeln sich andere Symptome oftmals schleichend nach Wochen oder Monaten. Insbesondere die Kombination von paradoxer Hyperalgesie und konsekutiver Erhöhung der Opioiddosis vermag psychische Veränderungen mit Antriebslosigkeit, Halluzinationen, Inappetenz, Erschöpfung, Schlaflosigkeit, nächtlichen Atemstörungen und körperlichem Abbau zu generieren [6]. Diese Symptomkonstellation, von uns im Folgenden als chronische Opioidintoxikation bezeichnet, erschwert, behandelbare Schmerzursachen zu erkennen, und begünstigt potenziell lebensbedrohliche Fehldiagnosen, falls infolge inadäquater Diagnostik der körperliche Verfall fälschlich als Zeichen einer beginnenden Sterbephase bei inkurabler Tumor‑, Herz- oder Lungenerkrankung interpretiert wird [5]. Genannt sei als Beispiel der Fall eines Patienten, der wegen therapieresistenter Schmerzen unter Fentanyl-Spray bei einem angeblich metastasierten Pankreaskarzinom zur palliativen Sedierung eingewiesen wurde. Nach einem Opioidentzug stellten sich kurativ behandelbare Bauchwandhernien als Schmerzursache heraus. Er lebte jahrelang ohne Rezidiv weiter [5]. Die Klinik der chronischen Opioidintoxikation ist facettenreich und abhängig von Grund- und Begleiterkrankungen. Bei Lungenerkrankungen kann sich eine schwere Ateminsuffizienz entwickeln, in anderen Fällen stehen scheinbar therapieresistente Schmerzen im Vordergrund, oft kombiniert mit kognitiven Einschränkungen und Zeichen der Abhängigkeit wie dem Einfordern höherer Dosen oder einer parenteralen Applikation, wie der folgende Fallbericht demonstriert.

## Fallbericht

Die seit Kurzem allein lebende fast 80-jährige Patientin wurde vom ambulanten Palliativteam unter der Verdachtsdiagnose eines Tumorrezidivs bei nicht beherrschbaren Schmerzen unter kontinuierlicher subkutaner Applikation von 150 mg Morphin/Tag (orale Morphinäquivalenzdosis [MÄq] 300–450 mg/p.o.) notfallmäßig in ein stationäres Hospiz eingewiesen.

Ihre Stimmung bei Aufnahme war bedrückt und sie äußerte explizit einen Sterbenswunsch. Ihre Wohnung habe sie aufgegeben, sie wisse, dass sie nun bald sterben würde. Sie klagte weder über Luftnot noch Schmerzen, jedoch über Inappetenz und Obstipation. Dagegen sei der Schlaf besser geworden, seitdem sie Morphin als Dauerinfusion erhalte.

Eineinhalb Jahre zuvor war ein kleines isoliertes und nicht metastasiertes Merkel-Zell-Karzinom im Bereich der rechten Kniekehle im Gesunden entfernt worden. Wegen diffuser Muskel- und Gelenkschmerzen mit Schwerpunkt auf der Seite der operierten Extremität erhielt sie schon länger 100 mg Tapentadol/Tag (MÄq 20 mg). Ein Jahr später zeigte sich bei intensiver Abklärung in einer Universitätsklinik kein Tumorrezidiv, wohl aber eine kompensierte chronische Niereninsuffizienz Stadium III mit einer glomerulären Filtrationsrate (GFR) von 21 ml/min, eine Kardiomyopathie (NYHA-Stadium II), zudem eine chronische Anämie mit Hämoglobinwerten von 7 bis 8 g/dl bei klinischem Verdacht auf eine erosive Gastritis, deren Abklärung die Patientin seinerzeit ablehnte, weil sie überzeugt war, dass sie an einem Rezidiv ihrer Krebserkrankung leide. Darauf führte sie auch ihre Schmerzen zurück. Hausärztlich wurde die Tumordiagnose auf Verlangen der Patientin nicht abgeklärt, die analgetische Therapie wurde umgestellt, da Tapentadol in Leitlinien bei Tumorschmerzen nicht empfohlen wird. Sie erhielt transdermales Fentanyl mit Patches von 100 µg/h (MÄq 240 mg/p.o.). Nach zwei Monaten wurde die Patientin an ein SAPV-Team (spezialisierte ambulante Palliativversorgung) überwiesen, nachdem sich der Allgemeinzustand bei zunehmender Immobilität und fehlendem Lebensmut deutlich reduziert hatte. In den folgenden 3 Monaten nahmen Schwäche und Schmerzen zu. Zehn Tage vor der stationären Aufnahme im Hospiz wurde trotz der bekannten Niereninsuffizienz auf Morphin zur kontinuierlichen subkutanen Infusion (Tagesdosis: ca. 150–180 mg, entsprechend 300–480 MÄq/p.o.) umgestellt, da sie bei nächtlichen Schmerzkrisen mehrfach von subkutanen Morphingaben profitiert habe und mit der Möglichkeit, das Morphin selbständig bei Bedarf zu verabreichen, sehr zufrieden sei. Der Arzt des SAPV-Team erklärte auf Nachfrage, er habe sich auf die hausärztlichen Angaben bezüglich der Tumordiagnose und des Verlaufs der Erkrankung verlassen.

Die körperliche Untersuchung der anämischen Patientin ergab wie auch zuvor andernorts keinen Hinweis auf ein lokales oder systemisches Tumorrezidiv wie vergrößerte Lymphknoten. An der Bauchdecke bestand um die Injektionskanüle ein Abszess, nach dessen operativer Sanierung die Morphindosis schrittweise auf 100 mg/24 h subkutan (MÄq 200–300 mg/p.o.) verringert wurde. Die Patientin wurde darunter deutlich vigilanter und es waren erstmals Perspektivgespräche möglich, in denen der Patientin vermittelt werden konnte, dass kein Tumorrezidiv vorliege und sie das Hospiz zeitnah verlassen könne und müsse. Ihr wurden diverse Kontakte zur Nachsorge und Unterstützung angeboten. Obwohl sich ihr Allgemeinzustand deutlich gebessert hatte, war die Patientin tief erschüttert. Denn ihre wirtschaftliche und soziale Situation erschien ihr ausweglos durch die Wohnungsauflösung, die in der Gewissheit eines baldigen Todes erfolgt war. In den Folgetagen entwickelten sich schmerzhafte Hauteffloreszenzen an den Beinen und Händen. Unter der Verdachtsdiagnose einer Immunvaskulitis stimmte die Patientin der Verlegung in eine Fachklinik zu, die die bereits initiierte Kortisontherapie fortsetzte. Sie erlitt dort trotz 20 mg Omeprazol/Tag eine Magenblutung auf dem Boden einer jetzt erstmals endoskopisch gesicherten schweren und vermutlich schon lange bestehenden erosiven Gastritis mit ösophagealer Beteiligung, die auch die Anämie hinreichend erklärte. Nach Ablehnung jeder weiteren Therapie durch die wache und voll orientierte, einwilligungsfähige Patientin wurde sie in das Hospiz zurückverlegt und verstarb dort nach wenigen Tagen an einer erneuten Ulkusblutung, deren Behandlung sie explizit erneut ablehnte.

Auch in diesem Fallbericht finden sich als Leitsymptome einer chronischen Opioidintoxikation scheinbar therapieresistente Schmerzen in Kombination mit Zeichen der Opioidabhängigkeit, hier die von der Patientin eingeforderte invasive Applikation der Opioide zur Behandlung von Schlafstörungen. Anders als sonst beschrieben [5, 7], ging die Fehldiagnose eines metastasierten Tumorleidens primär nicht von den Ärzten aus, sondern von der Patientin selbst, möglicherweise erklärbar aus ihrer sozialen Situation. Aber der Zusammenhang mit der Opioidtherapie ist wahrscheinlich, wie die Verbesserung der Vigilanz und die erstmalige Akzeptanz einer kurativen Behandlung nach der Opioidreduktion belegen. Typischerweise kam es davor unter den Opioiden zu einem progredienten körperlichen und seelischen Verfall, der die behandelnden Ärzte dazu verleitete, an diese Tumordiagnose trotz fehlender Befunde zu glauben. Aber wie schon vor fast 30 Jahren in einem ähnlichen Fall analysiert: Wenn erst einmal die Überzeugung besteht, dass es sich um ein nicht behandelbares Leiden handelt, entsteht daraus eine potenziell fatale Tendenz zur „self-fulfilling prophecy“ [7]. Also wurden Symptome wie die gastritisassoziierte Anämie, die kurativ behandelbar gewesen wäre, dieser Fehldiagnose zugeordnet, andernfalls wäre vielleicht der fatale und letale Verlauf zu verhindern gewesen.

Der Fall unterstreicht erneut die Forderung, bei starken Schmerzen unter Opioiden alle Diagnosen zu reevaluieren. Im Zweifel sollte bei dieser Konstellation auch aus diagnostischen Gründen die Opioiddosis reduziert werden. Diese bei chronischen Schmerzen bewährten Regeln gelten auch für Palliativpatienten, bevor man den Schmerz für nicht oder nur invasiv behandelbar erklärt und die Patienten zum Sterben in ein Hospiz verlegt. Wir publizieren den Bericht auch in der Hoffnung, dass die Diagnostik und Therapie der chronischen Opioidintoxikation zukünftig auch in der Weiterbildung und in Leitlinien ein höheres Gewicht erhält.

## References

[1] AWMF -Erweiterte S3-Leitlinie. Palliativmedizin für Patiente mit einer nicht-heilbaren Krebserkrankung Langversion 2.2 – September 2020 AWMF-Registernummer: 128/001OL (Aufruf 1.9.2025)

[2] Biesbrouck T, Jennes DA, Van Den Noortgate N, De Roo ML (2024) Pharmacological treatment of pain, dyspnea, death rattle, fever, nausea, and vomiting in the last days of life in older people: A systematic review. Palliat Med 38(10):1088–1104. 10.1177/02692163241286648 39390791

[3] Gupta D, Mishra S, Bhatnagar S (2007) Somatization disorder, a cause of difficult pain: a case report. Am J Hosp Palliat Care 24(3):219–223. 10.1177/1049909106298395 17601846

[4] Masman AD, van Dijk M, Tibboel D, Baar FP, Mathôt RA (2015) Medication use during end-of-life care in a palliative care centre. Int J Clin Pharm 37:767–775. 10.1007/s11096-015-0094-3 25854310 PMC4594093

[5] Michel-Lauter B, Bernardy K, Schwarzer A, Nicolas V, Maier C (2013) Abhängigkeitssyndrom und Hyperalgesie durch Opiode bei kurativ behandelbaren Schmerzen: Ein Fallbericht zu einem scheinbaren Palliativpatienten. Schmerz 27:506–512 24005817 10.1007/s00482-013-1353-7

[6] Schofferman J (1993) Long-term use of opioid analgesics for the treatment of chronic pain of nonmalignant origin. J Pain Symptom Manage 8(5):279–288. 10.1016/0885-3924(93)90156-p 7525744

[7] Taube AW, Jenkins C, Bruera E (1997) Is a ”palliative“ patient always a palliative patient? Two case studies. J Pain Symptom Manage 13(6):347–351. 10.1016/s0885-3924(97)00080-8 9204655

